# Supplementary material for: Empirical Bayes functional models for hydrogen deuterium exchange mass spectrometry
Source: Commun Biol. 2022 Jun 15;5:588. doi: 10.1038/s42003-022-03517-3 (PMC9200815; doi:10.1038/s42003-022-03517-3)
Supplement: Supplementary file 2 — Supplementary Material [file 42003_2022_3517_MOESM2_ESM.pdf]

Supplementary material: Empirical Bayes functional models for hydrogen deuterium exchange mass spectrometry

Oliver M. Crook * [1], Chun-wa Chung[2], and Charlotte M. Deane[1]

[1] *Department of Statistics, University of Oxford, Oxford, OX1 3LB, UK*
[2] *Structural and Biophysical Sciences, GlaxoSmithKline R&D, Stevenage, SG1 2NY, UK*

May 19, 2022

Supplementary Material

Supplementary Note 1

Here, we plot the peptide that had significantly altered deuterium kinetics. For some dAbs no such cases were found. The dAbs are subscripted by their molar concentration with respect to HOIP-RBR. No significant results are reported for dAb3, dAb6, dAb14

*oliver.crook@stats.ox.ac.uk

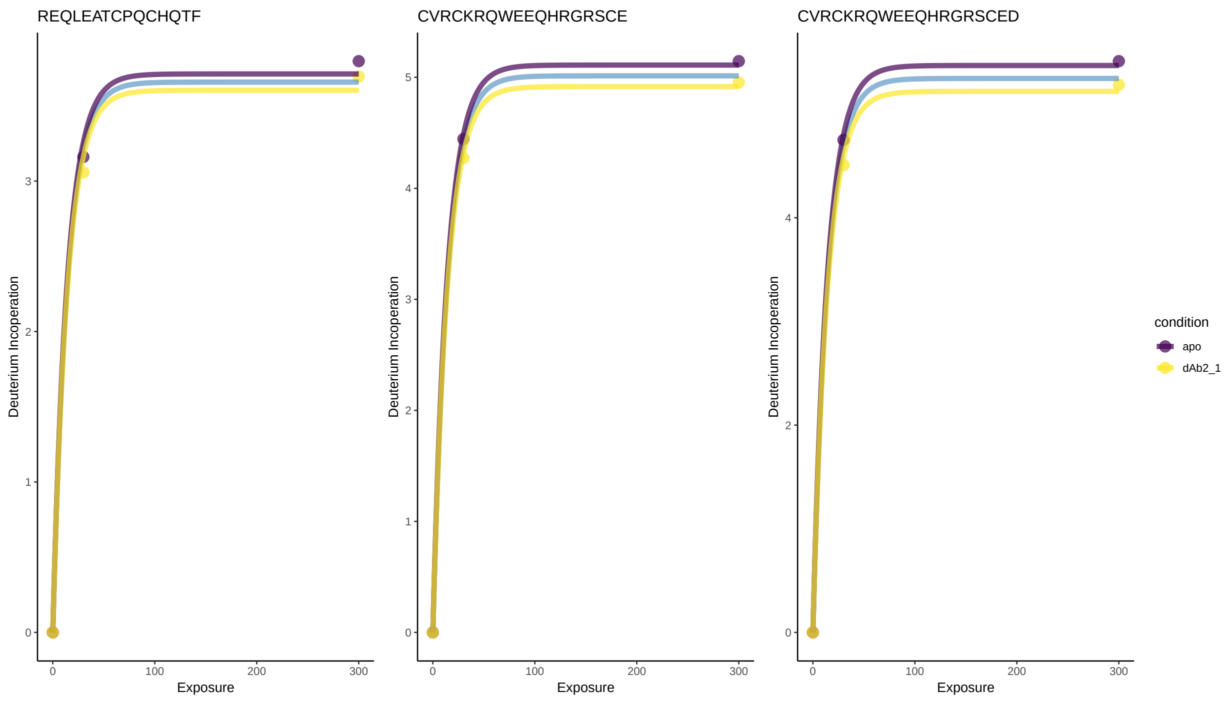

Figure S1: **dAb2$_1$.** b = 0.07, FDR < 0.05

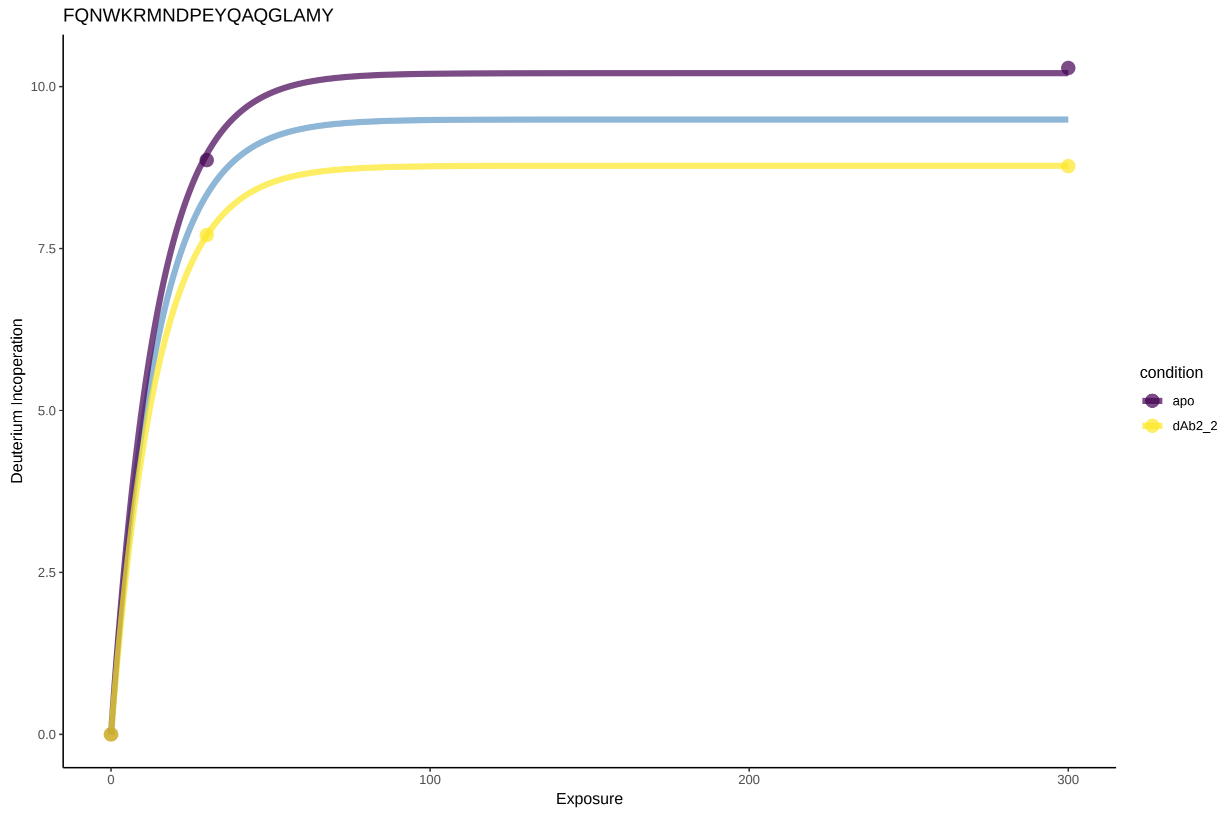

Figure S2: **dAb2$_2$.** b = 0.07, FDR < 0.05

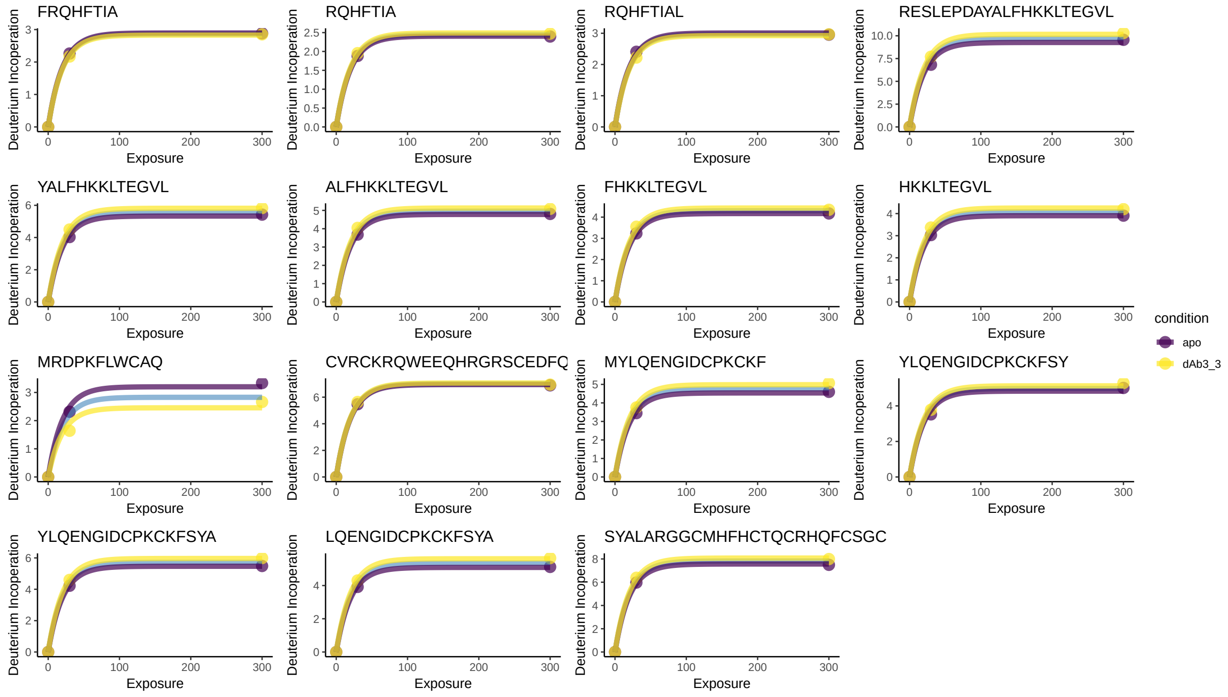

Figure S3: **dAb**18_3**.** b = 0.05, FDR < 0.05

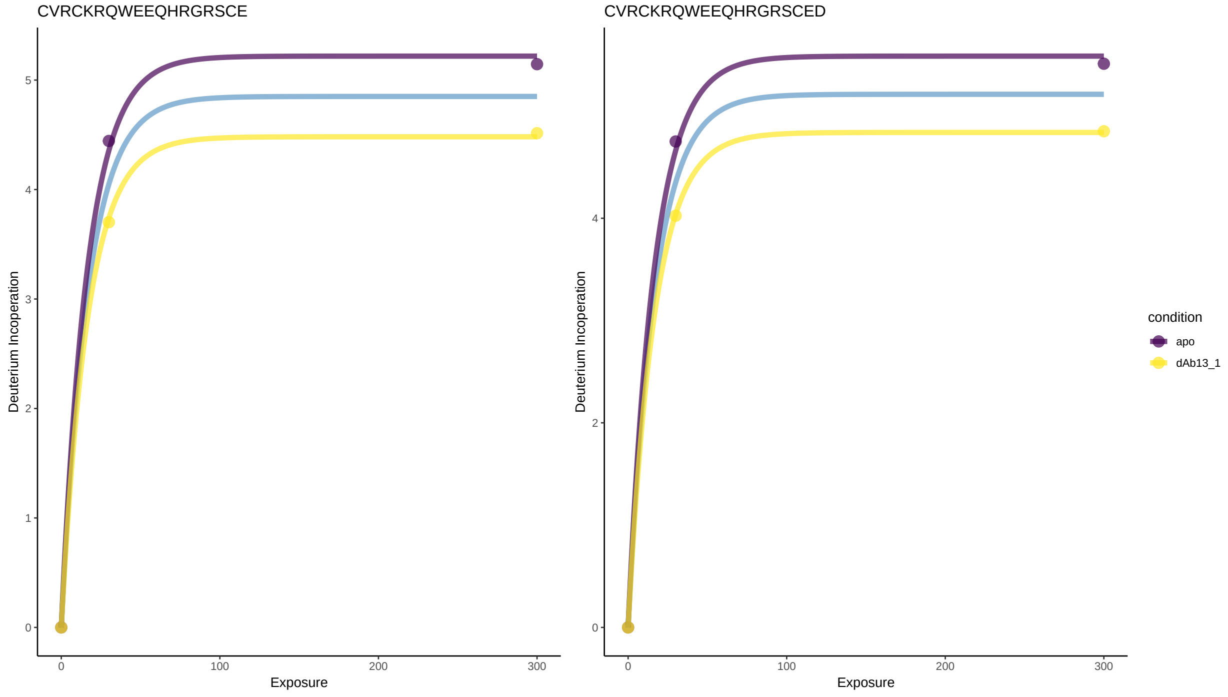

Figure S4: **dAb**13_1**.** b = 0.06, FDR < 0.05

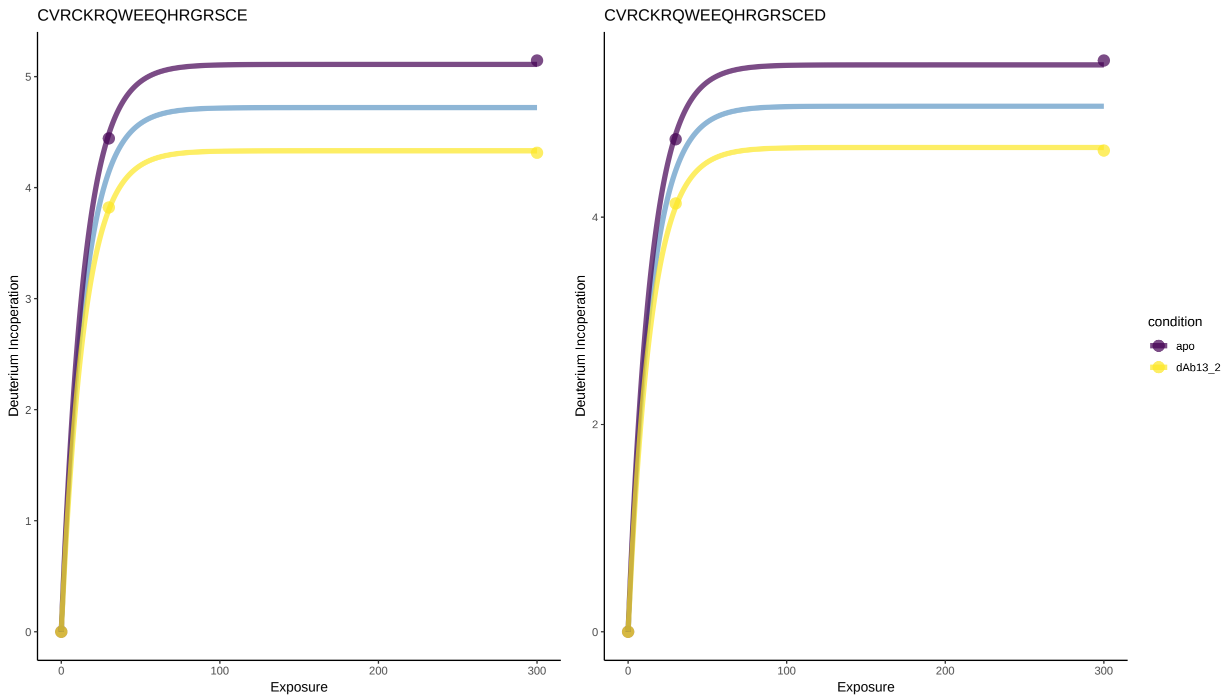

Figure S5: **dAb13$_2$.** b = 0.07, FDR < 0.05

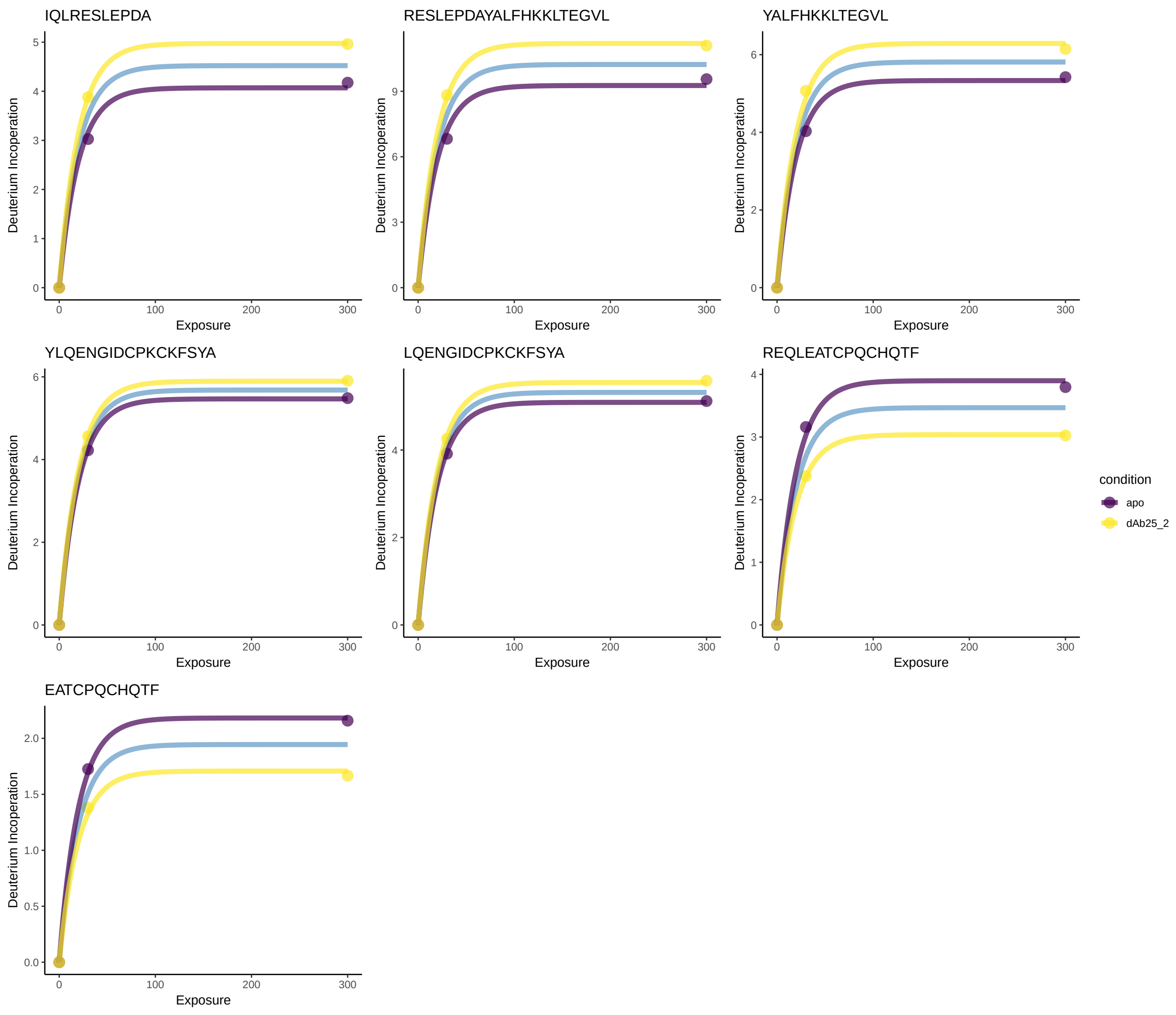

Figure S6: **dAb25$_2$.** b = 0.05, FDR < 0.05

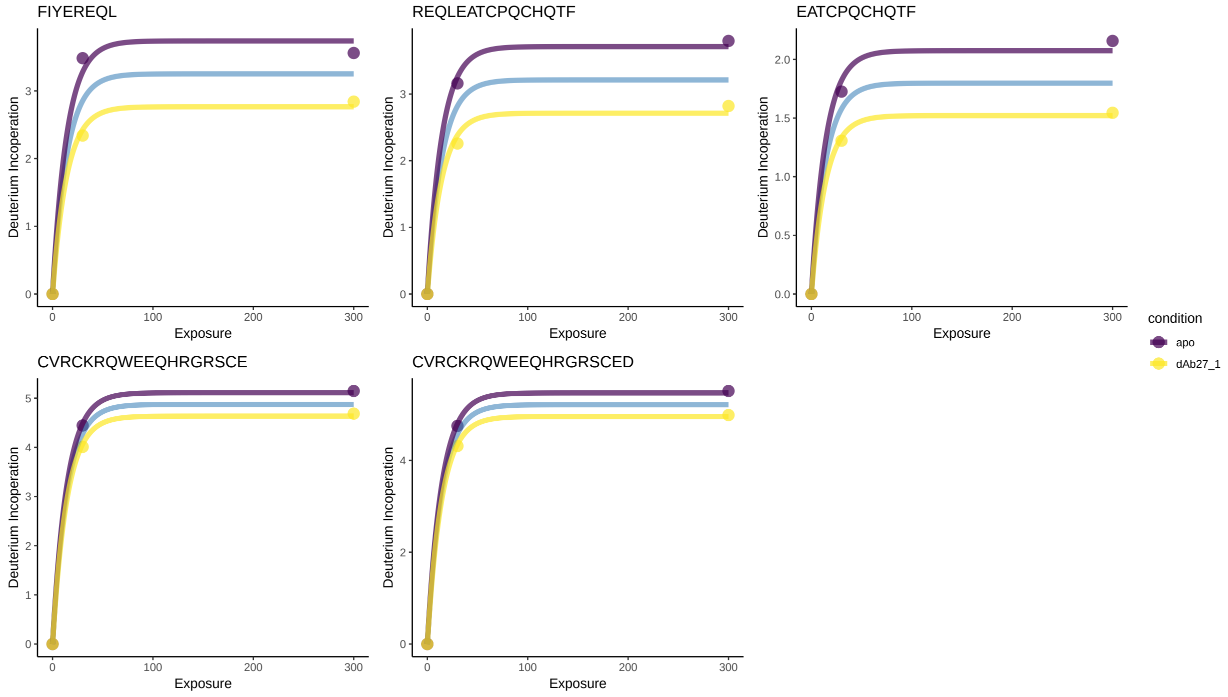

Figure S7: **dAb27$_1$.** b = 0.07, FDR < 0.06

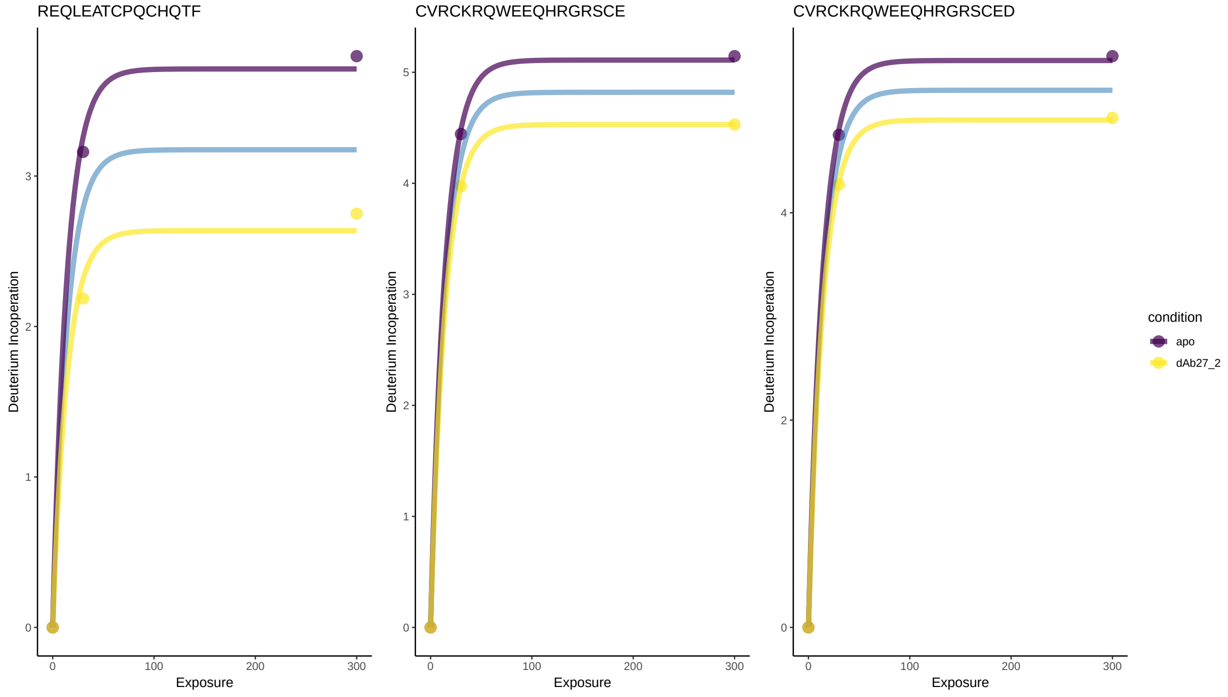

Figure S8: **dAb27$_2$.** b = 0.07, FDR < 0.05

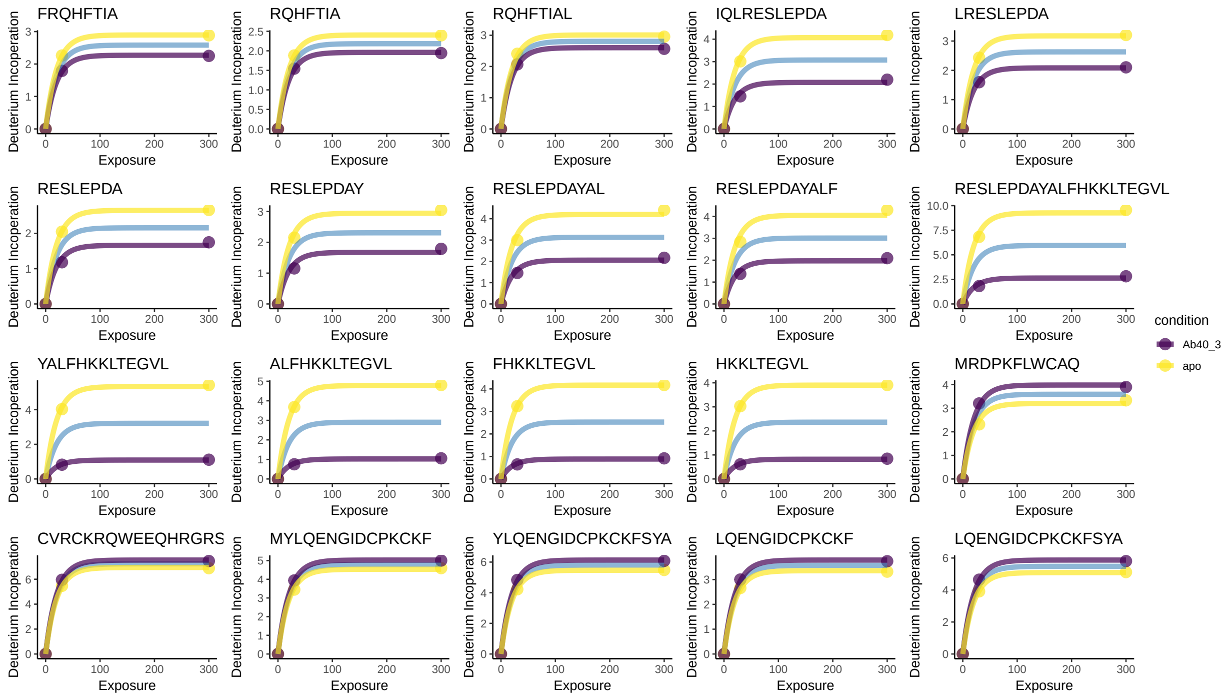

Figure S9: **dAb**40_3**.** b = 0.05, FDR < 0.05

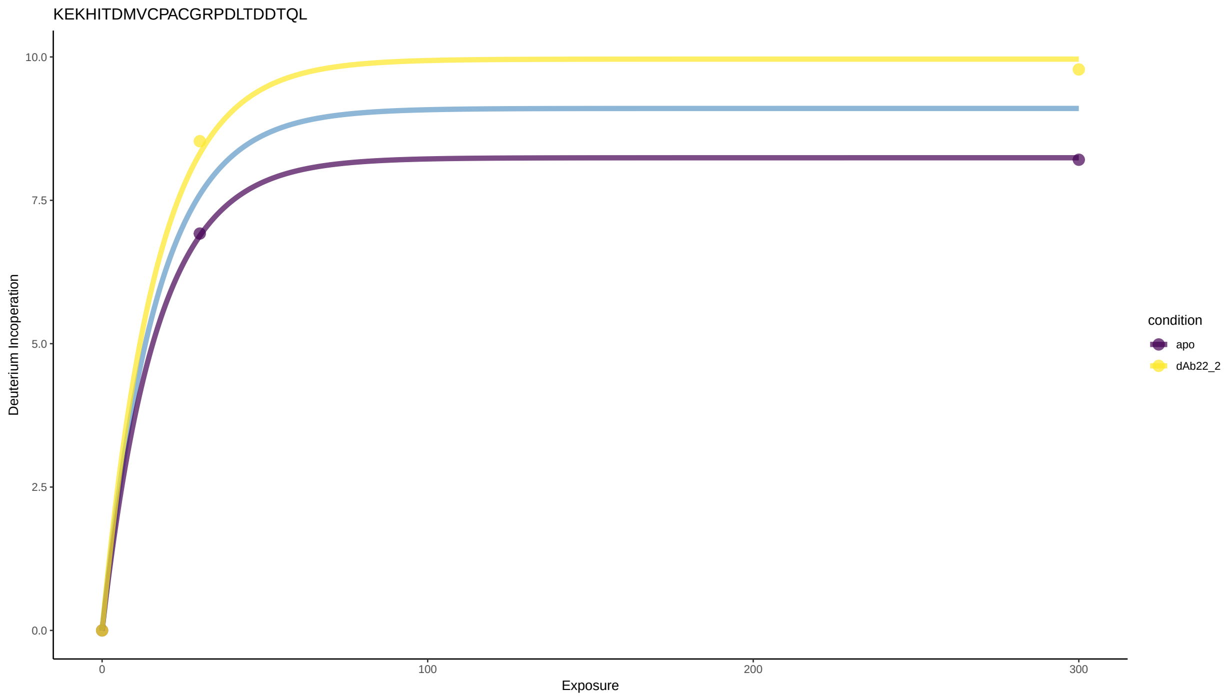

Figure S10: **dAb**22_2**.** b = 0.06, FDR < 0.05

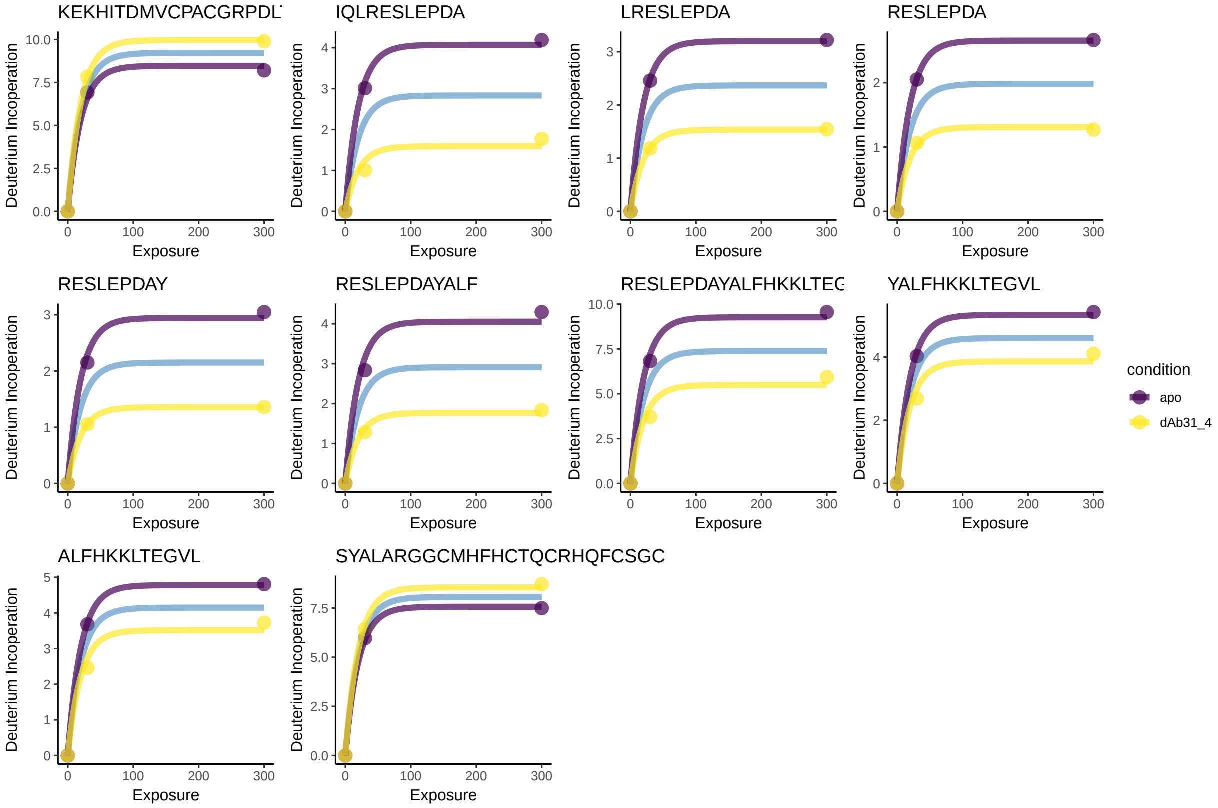

Figure S11: **dAb**31_4**.** b = 0.05, FDR < 0.05

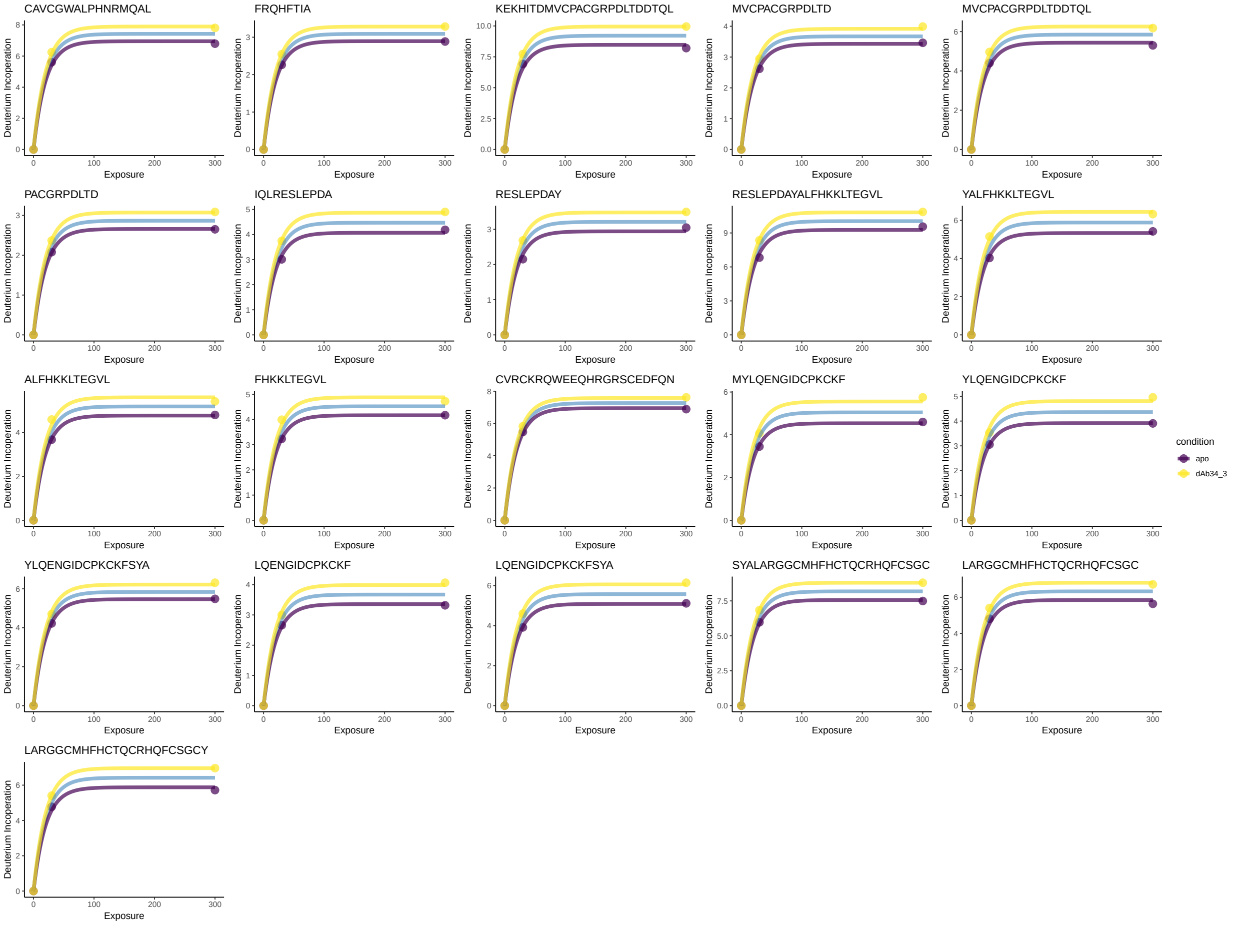

Figure S12: **dAb**34$_3$**.** b = 0.05, FDR < 0.05

Supplementary Note 2: Further visualisation of HOIP-RBR-dAB25

Alongside the Manhattan plot shown in the main text, we can plot significance on a standard peptide coverage plot. This allows us to obtain another global and spatial visualisation of the significantly altered peptides. This allows us to quickly identify support for our claims by examining whether overlapped peptides are also significantly perturbed, which can be tricky using a Manhattan plot. A number of factor can lead to an overlapping peptide not being declared significant and not declaring significance should not be considered evidence of the null.

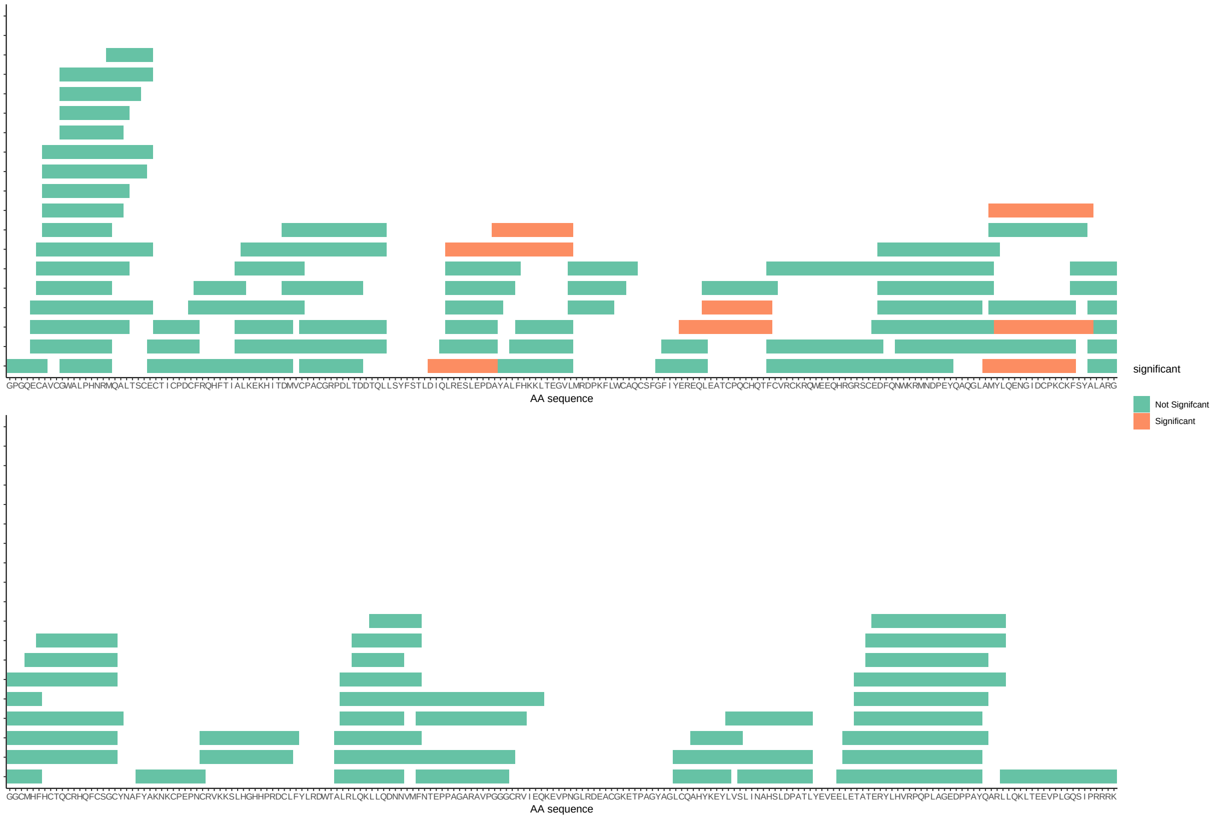

Figure S13: **Peptide coverage plot with significance overlaid for dAb25$_1$.**

Supplementary Note 3: Global visualisation of multi-antibody Epitope mapping

Alongside the functional uptake plots and peptide coverage plots, we can visualise residue level results by obtaining a score for each peptide. This is obtained using the harmonic mean of the p-values at each residue (see methods). We can then also plot all dAbs used in an experiment alongside each other.

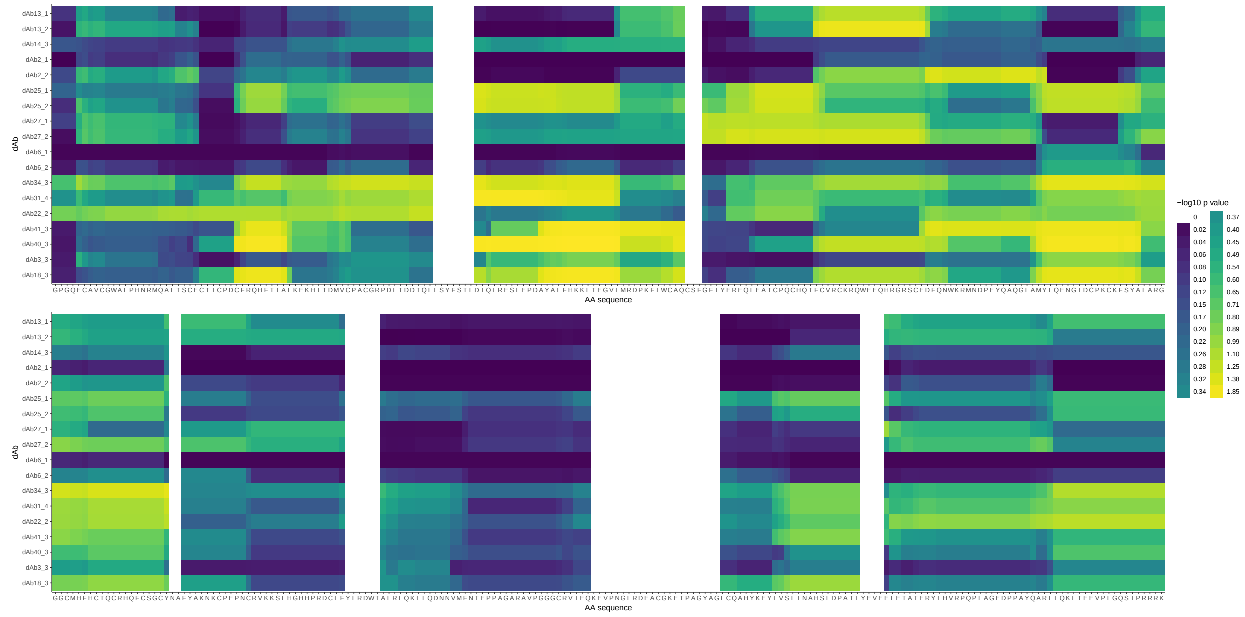

Figure S14: **Heatmap showing residue level significance for all antibodies.** Residue level visualisation plotted as a heatmap (see methods), for all dAbs used in the experiment.

Supplementary Note 4: Support for other models within "hdxstats"

The accompanying "hdxstats" package also supports further functional modelling strategies. For example, the logistic and Weibull models can be replaced with more exotic models which may be more appropriate to the application. Biphasic exchange may support sums of logistic model, such as:

$$\mu(t) = a(1 - \exp(-bt)) + c(1 - \exp(-dt)). \tag{1}$$

The statistical methodologies detailed in the main article still apply by simply replacing the functional model for the one above. Furthermore, the package also supports comparison of different models using statistical methods such as the log-likelihood. For demonstration, we fit the a sums of logistic model to an example peptide from the MBP dataset (see figure S15) and we show a comparison of this to the fit from the Weibull model (see figure S16)

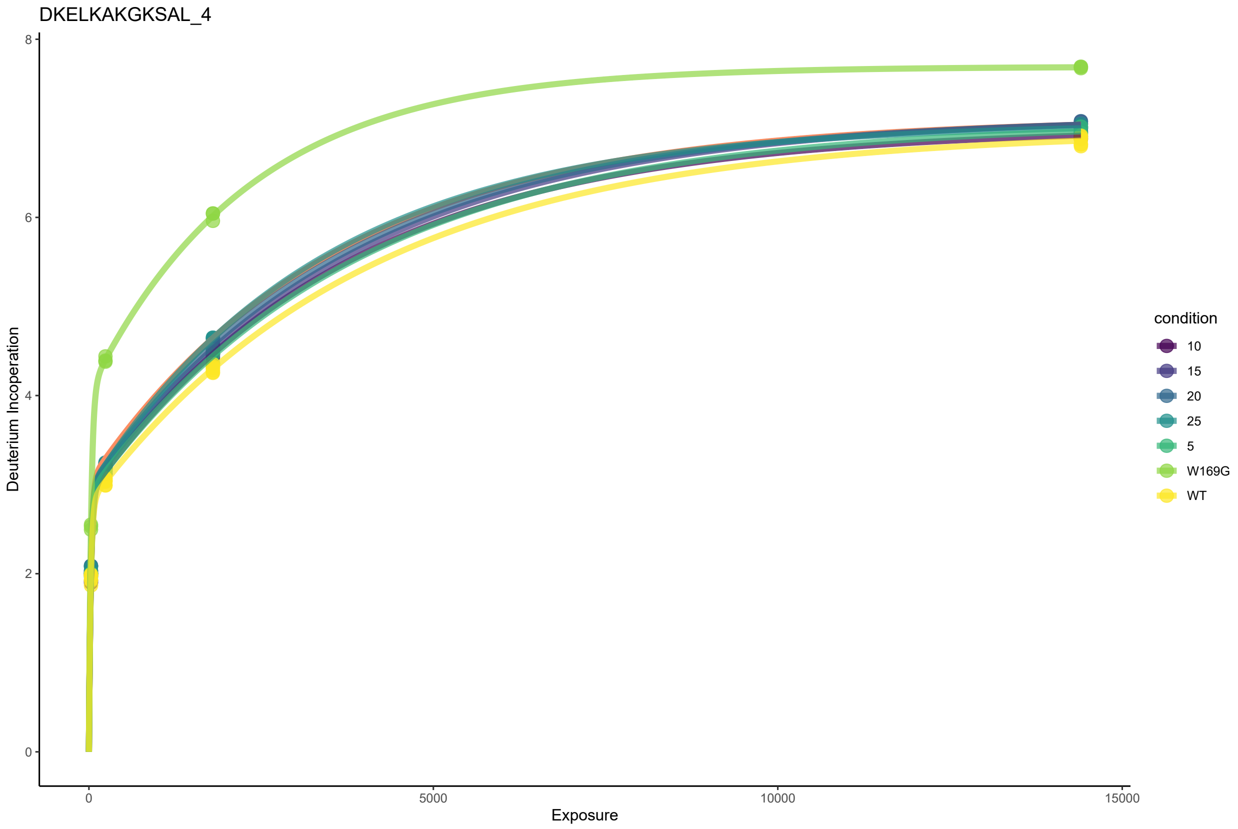

Figure S15: **Fitting sums of logistics to HDX-MS data** Functional models fit to HDX-MS kinetics data.

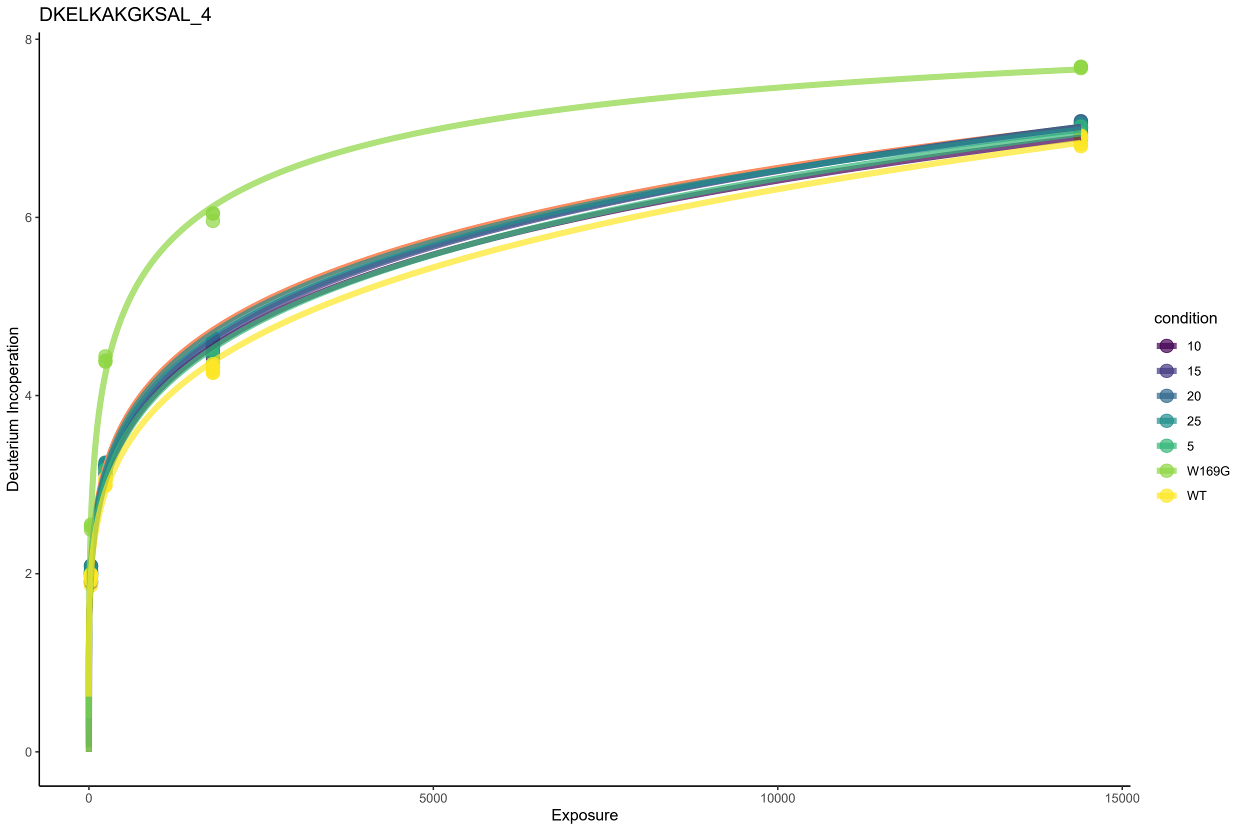

Figure S16: **Fitting the Weibull model to HDX-MS data** Functional models fit to HDX-MS kinetics data.

Supplementary Note 5: Further simulations

To test the effect of noise on the various statistical approaches, we varied the standard deviation of the residual noise. We perform the following additional simulation scenarios:

- Standard deviation = 0.1 and 3 replicates (simulation 7)
- Standard deviation = 0.2 and 3 replicates (simulation 8)
- Standard deviation = 0.5 and 3 replicates (simulation 9)
- Standard deviation = 0.1 and 2 replicates (simulation 10)
- Standard deviation = 0.2 and 2 replicates (simulation 11)
- Standard deviation = 0.5 and 2 replicates (simulation 12)

Our simulations demonstrate that even in very noisy data our functional model achieves the highest performance (see figure S17).

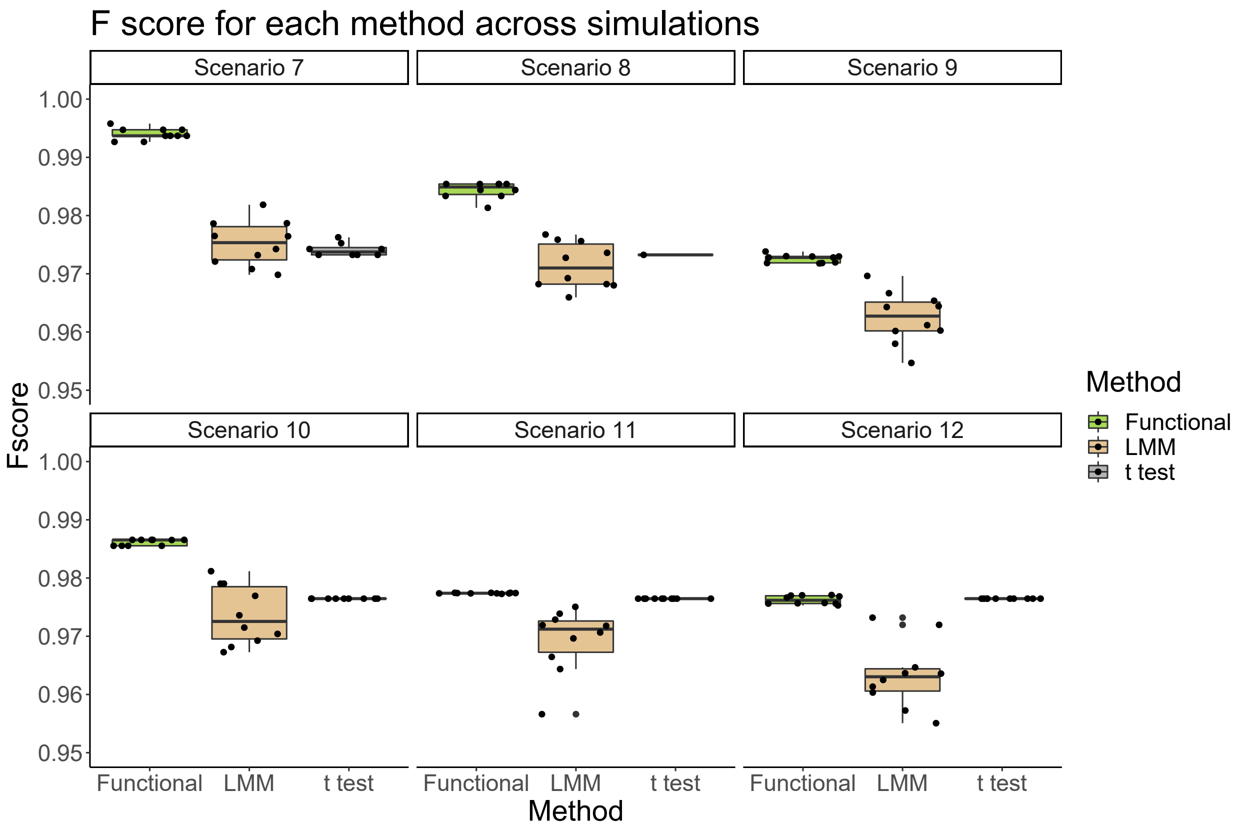

Figure S17: **Further simulations demonstrating the effect of noise** Simulation study for HDX data showing improved performance of our proposed functional method (functional) for linear mixed models (LMM) and the t-test (t test). These simulation encompass scenarios with differing standard deviation and replicates. Failed computations not shown.

Supplementary Note 6: Sensitivities in simulations

To examine false-negatives, we compute the sensitivity (true positive rate) of all the approaches across all the simulation settings. We found that the linear mixed model (LMM) approach has the highest sensitivity across all the approaches. However, this is at the cost of an unacceptable inflation of false-positives (see main text.). The sensitivity of our approach could be improved by switching to a fully Bayesian approach.

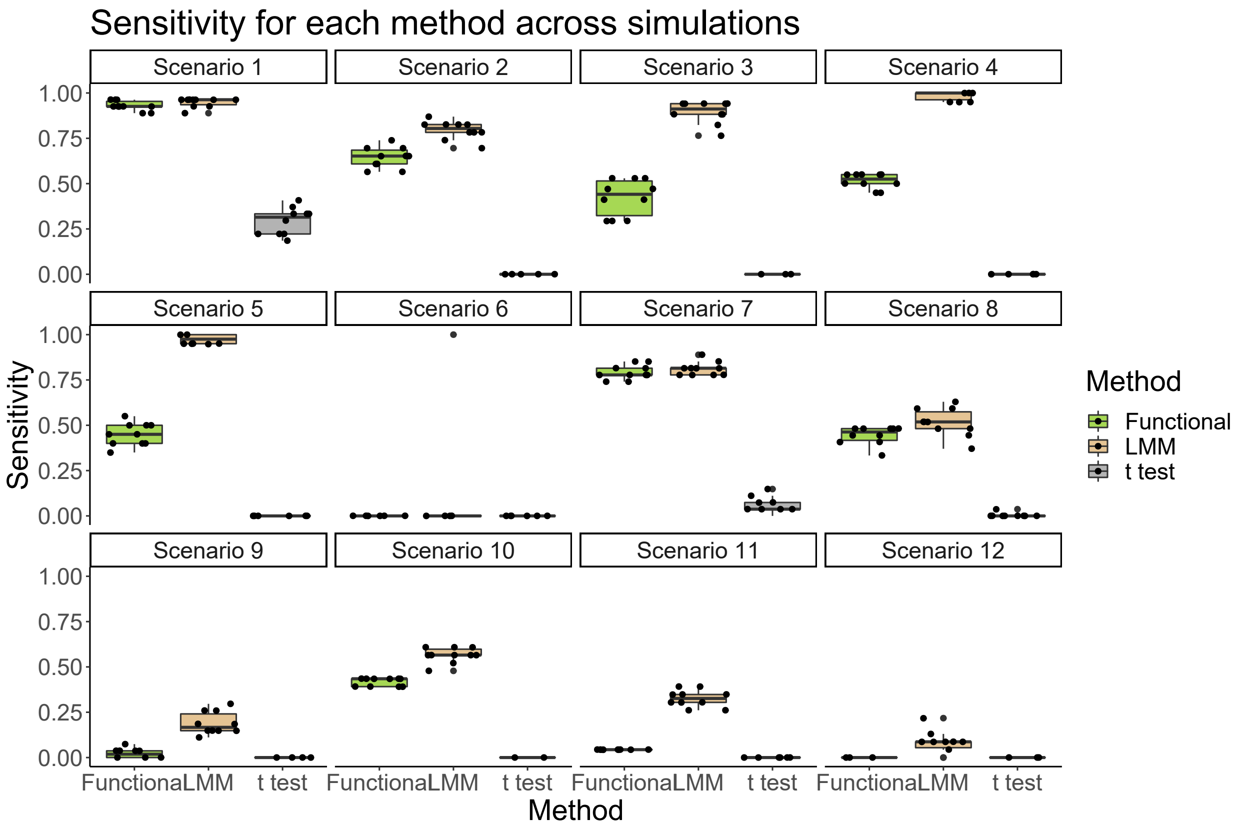

Figure S18: **Examining sensitivities in each approach through simulations** Simulation study for HDX data showing improved performance of our proposed functional method (functional) for linear mixed models (LMM) and the t-test (t test). These simulation encompass scenarios with differing standard deviation, replicates, time-points and missing values. Failed computations not shown.
